# Two restraining devices in connection to surgical castration with or without local anesthesia: effects on piglet stress

**DOI:** 10.1186/s40813-025-00428-7

**Published:** 2025-04-15

**Authors:** Ann-Helena Hokkanen, Mathilde Coutant, Mari Heinonen, Marianna Norring, Magdy Adam, Claudio Oliviero, Tiina Bergqvist, Anna Valros

**Affiliations:** 1 Department of Production Animal Medicine, Faculty of Veterinary Medicine, University of Helsinki, Helsinki, Finland; 2 Research Centre for Animal Welfare, Faculty of Veterinary Medicine, University of Helsinki, Helsinki, Finland; 3 Department of Animal and Veterinary Sciences, Aarhus University, Tjele, Denmark

**Keywords:** Piglets, Handling, Castration bench, Procaine with epinephrine, Castration, Pain, Behavioral reactions, Animal welfare, *Sus scrofa*, Management

## Abstract

**Background:**

Surgical castration causes severe pain to young piglets. In addition, piglets experience intense stress from handling and restraining during the procedure. Furthermore, piglets must be restrained twice when receiving local anesthesia before castration, and the injections are painful. Unfortunately, strategies to reduce piglets’ stress during handling, local anesthetic injections, and painful procedures are limited and poorly understood. Thus, we randomized 179 male piglets aged 3 to 4 days to be restrained with either a commonly used commercial tubular bench with a dorsal restraining posture or a custom-made castration rack with a vertical restraining posture. Piglets received local anesthetic or sham injections (mimicking local anesthesia but without skin penetration) 10 min before castration. We then compared the effect of these devices on the piglets’ behavioral reactions, vocalizations, and body temperatures at the following four times: when restrained for the first time, when given local anesthetic injections or receiving sham injections, during the second time restrained, and during castration.

**Results:**

Piglets were given higher mean reaction scores when restrained in the tubular bench than the castration rack. Piglets showed differing vocalization patterns in the two devices, with more grunts and screams in the castration rack and more squeals in the tubular bench. Moreover, local anesthetic injections resulted in higher mean reaction scores and longer vocalizations than sham injections during the injections/sham injections but reduced these measures during castration. After castration, the skin temperature was higher in sham-injected piglets than in piglets castrated with local anesthesia, irrespective of the restraining device used.

**Conclusions:**

Local anesthesia reduced piglets’ reaction scores and vocalizations during castration. However, receiving local anesthesia was painful. Based on piglet behavior, restraining in a tubular bench was more aversive than in the castration rack. However, the devices affected the proportions of the different vocalization types during local anesthetic injections and castration in a manner we cannot fully explain. As the restraining method affects piglets’ behavior, it may influence pain assessment during local anesthetic injections and evaluation of local anesthesia efficacy during castration. Thus, less stressful methods to handle piglets are needed.

**Supplementary Information:**

The online version contains supplementary material available at 10.1186/s40813-025-00428-7.

## Background

During surgical castration, piglets < 7 days of age are confined by holding or attaching them to a bench. Two parallel incisions to the skin over the testicles are performed with a scalpel, both testicles are extracted from the scrotum, and the spermatic cords are severed [1]. Despite the brevity of the procedure, it causes considerable stress [2–4], extensive tissue damage [5, 6], and severe pain both during [7, 8] and after the surgery [9, 10]. When piglets are castrated without any pain alleviation, they will scream, try to escape the procedure, and swing their legs vigorously [11]. Pain experienced early in life may have many long-lasting adverse effects on pig welfare [12, 13] and should, therefore, be reduced to minimum.

Although the need for alternatives to surgical castration is widely acknowledged, implementing these methods takes time. Accordingly, effective pain alleviation during castration is needed [14, 15]. Currently, in Finland, qualified persons are allowed to castrate male piglets < 7 days of age with an open surgical method without tearing the tissues. Before castration, piglets must receive a non-steroidal anti-inflammatory drug (NSAID). Since January 2024, people who have completed training and passed the qualification test are allowed to apply local anesthesia to the piglets before castration. By 2027, the use of local anesthesia before castration will be mandatory [16]. However, while using pain alleviation with an NSAID and a local anesthetic will reduce piglets’ reactions to castration, they seldom eliminate pain entirely during or after castration [3, 4, 10, 17, 18].

In addition to castration pain, piglets experience intense stress from handling and restraining during the procedure [19]. Furthermore, piglets must be restrained twice when they receive local anesthesia before castration. First, the piglets are handled for NSAID administration and injecting local anesthesia. Then, a waiting period of 5 to 30 min is needed for the local anesthetic agent to take effect [20]. The piglets are then handled again for castration.

Piglet behavioral reactions and vocalizations have been previously studied in connection to stress and pain caused by local anesthetic injections and castration [2, 3, 11, 19, 21, 22]. Moreover, skin temperature changes can indicate stress and pain [11, 23–25]. However, to our knowledge, there are no studies on how piglets should be held during the administration of local anesthesia to minimize stress. Only one study has explored how piglets perceive different restraining devices during castration itself [22]. One person can perform castration without a restraining device. However, the piglet needs to be restrained by an additional person or a restraining device when local anesthesia is administered to allow the use of both hands for the procedure itself. According to the authors’ observations, piglets are calmer when restrained in the lap than when using a restraining device, but this, of course, increases labor costs. A tubular restraining device is commonly used, where the piglets are restrained in a dorsal position. Based on the authors’ experience, this method causes a very strong reaction in piglets. In addition to this being a sign of potentially intense stress in the piglets, behavioral reactions due to handling stress may compromise the evaluation of pain needed to assess the efficacy of local anesthesia and other pain-mitigating strategies applied at castration. Moreover, the tubular restraining device is difficult to clean between litters and time-consuming to use. There is thus a need to develop and evaluate alternative restraining methods for piglets. One possible alternative to the tubular restraining device is a custom-made castration rack, where the piglets are restrained in a vertical position and hung from their groins with their heads down while held in place by the handler’s body. The practical benefit of this device is that it poses fewer hygiene challenges and is perceived as easier and faster to use than a tubular restraining device. However, in both devices, the piglets are restrained in unnatural postures.

The primary objective of this study was to compare the effect of two different restraining devices, a commercially available tubular castration bench (TUBE) and a custom-made castration rack (HANGING), on piglets’ stress during handling, administration of local anesthesia, and surgical castration. The secondary aim was to assess whether the restraining device affects the clarity of behavioral differences between piglets during the administration of local anesthesia versus sham injections and between piglets castrated with or without local anesthesia. To that extent, we evaluated the animals’ behavioral reactions, vocalizations, and skin temperature. Moreover, we studied the time required to restrain piglets, to administer local anesthesia, and to castrate the piglets in these devices.

## Methods

The University of Helsinki Viikki Campus Research Ethics Committee (17/2023) approved the study protocol. Written consent from the animal owners was obtained.

### Animals and housing

We conducted the study in the farrowing unit of a commercial piglet-producing farm in Southern Finland. The experiment day was a farm’s routine castration day in October 2023. In total, we included 179 male crossbred piglets (Yorkshire & Finnish Landrace x Tempo) from 23 litters.

The average parity of the sows was 3.4 (range 1–5), including one litter from a primiparous sow. The sows were fed daily 4 kg of a mixed ration (a farm-made feed mixture with energy 8.8 MJ, crude protein 150.7 g/kg, and crude fiber 57.9 g/kg). Water was available *ad libitum*.

All piglets were born during the same week and housed in similar conditions in two different farrowing rooms, each housing 45 sows in pens (3 m x 2.5 m) with partly slatted flooring. Sows were loose housed but confined in crates during the first days after farrowing. The crates were opened after piglet castration. The room temperature in the farrowing unit was 21.5 °C. The piglet nest had a solid concrete floor bedded with sawdust and a floor temperature of 38 °C. On the day of the study, the age of the piglets was 3 days in 22 litters and 4 days in one litter. Piglets were not ear tagged or marked, not tail docked, and their teeth were left intact. As the study was performed in connection with the normal farm routines, we were unable to weigh the piglets. However, we divided the piglets into two size groups (normal and small) after visually estimating their size, thus simulating how it would be done in practice on a pig farm. We included and randomly allocated to the different groups only piglets in sound general condition with completely descended testicles and free of overt anatomical malformations.

### Study design

#### Timeline

**Fig. 1:**
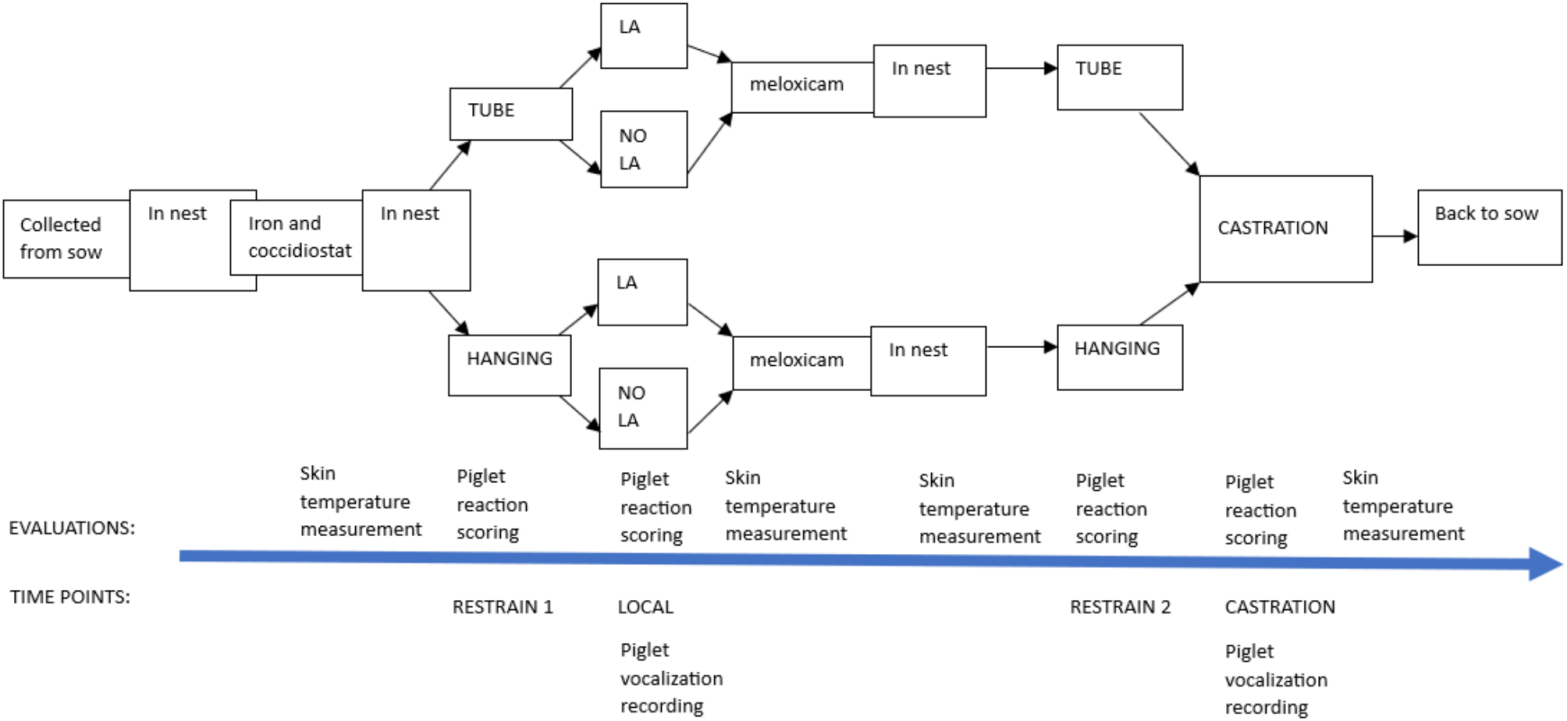
Timeline of the study investigating restraining devices and the use of local anesthesia in piglet castration. An injection containing iron and coccidiostat and an injection of meloxicam were administered to all study piglets. The piglets were restrained at two time points (RESTRAIN 1 and RESTRAIN 2) using either a tubular castration bench (TUBE) or a castration rack (HANGING). The piglets received either local anesthesia (LA) or sham injections (NO LA) at the timepoint LOCAL. Finally, surgical castration was performed at the timepoint CASTRATION. Between all procedures, the piglets stayed in their nests

The study timeline is presented in Fig. 1. An experienced farm employee (later referred to as the handler) performed piglet handling and castration. Initially, the handler carefully collected all the piglets and placed them in the piglet nest. Subsequently, she lifted one piglet at a time from the nest and injected a suspension of 45 mg toltrazuril (coccidiostat) and 200 mg gleptoferron (iron) (Forceris™, 1.5 mL, Ceva Animal Health A/S, Libourne, France) intramuscularly and returned the female piglets to the sow. She turned male piglets towards one of the researchers, who measured skin temperature and marked the piglet in numerical order with a text marker. Then, the handler put the male piglets back in the nest. From the nest, the handler picked piglets one by one unaware of the predetermined treatment order used to ensure all treatments were performed within each litter and to achieve a similar number of piglets within each treatment.

When all male piglets from the given litter were in the nest, the handler lifted the first male piglet (in numerical order) and fixed it into the restraining device (either TUBE or HANGING according to the predetermined order, described in more detail below under the heading *procedures*). Then, the handler fixed the piglet in place while a veterinarian approached the piglet and secured it in place in the device using his hands (TUBE) or his body (HANGING). At this stage, the two observers scored the reaction of the piglet (timepoint RESTRAIN 1). Then, the veterinarian administered either local anesthesia (LA) or sham injections (NO LA), and the observers scored the reaction to LA or NO LA (timepoint LOCAL). Subsequently, the handler took the piglet out of the device, injected the NSAID meloxicam (Metacam^®^ 5 mg/mL, injection solution for cattle and swine, Boehringer Ingelheim Pharma GmbH & Co. KG, Ingelheim am Rhein, Germany; target dose approximately 0.4 mg/kg body weight intramuscularly according to farm routine practice), turned the piglets towards the researcher for skin temperature measurement, and then returned the piglet to the nest. We handled all male piglets in the litter the same way, and video and audio recorded all procedures using an iPhone 11 pro, video HD (1080 pixels) 30 frames per second.

After all male piglets in the litter had been handled, there was a 10-minute interval between LA administration for the first piglet and starting castration, during which time we performed the same procedures described above for the next litter.

After the 10-minute waiting period, the handler took the first piglet (in numerical order starting at number one, i.e., the piglet that received LA first) from the nest, turned it to the researcher for skin temperature measurement, and then put the piglet in the restraining device according to its assigned treatment (TUBE or HANGING). The observers scored the reaction of the piglet (timepoint RESTRAIN 2). Then, the handler surgically castrated the piglet, and the observers scored the reaction of the piglet to castration (timepoint CASTRATION). Then, the handler took the piglet from the device, turned it towards the researcher for skin temperature measurement, and carefully put it back in the pen with the sow. At this point, the study ended for this individual piglet. We then performed the same procedures for the other piglets of the litter in numerical order.

#### Procedures

**Fig. 2:**
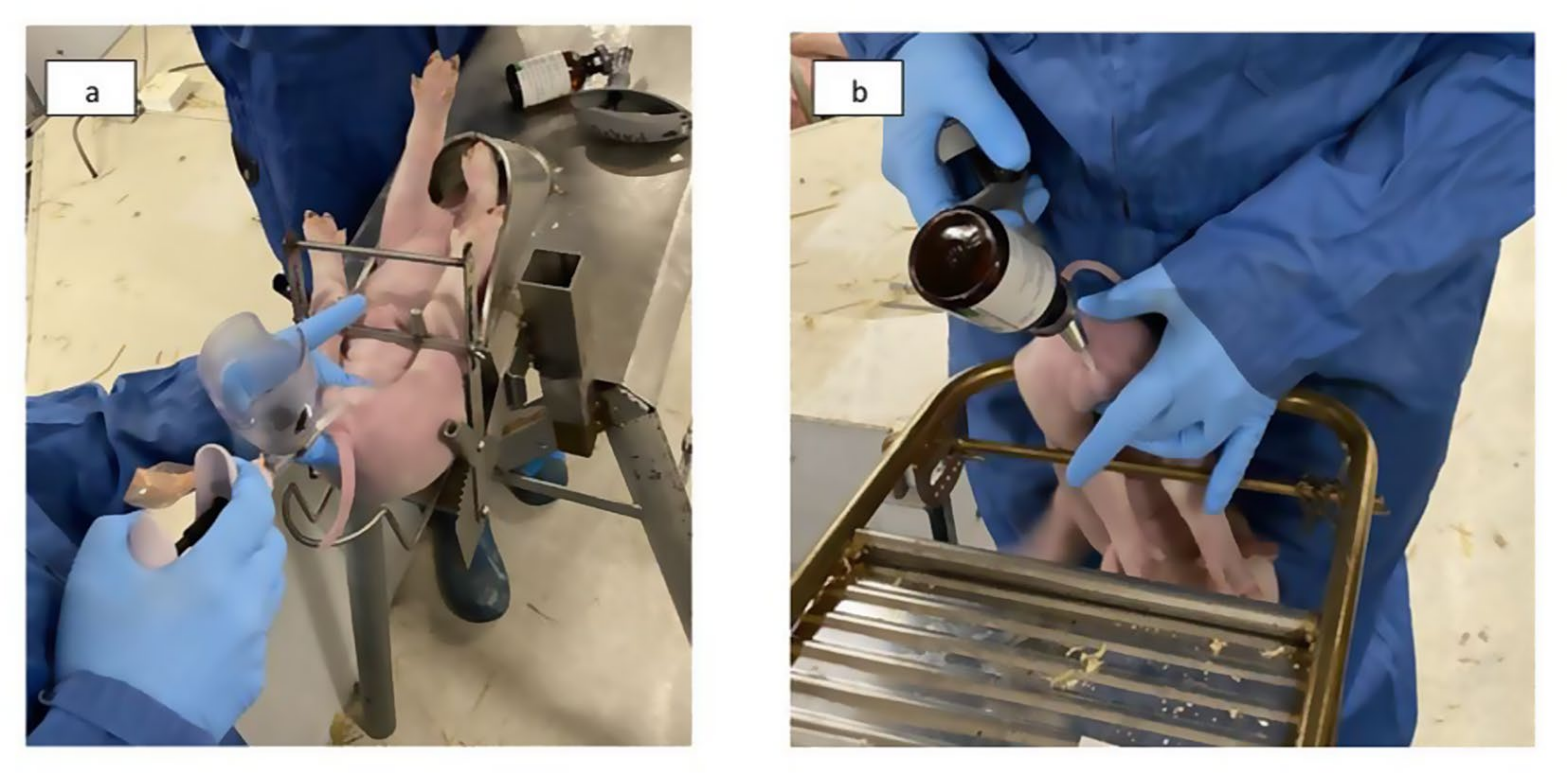
Piglet restraining devices used in the study. Piglets were restrained either with a tubular castration bench (Fig. 2a) or with a castration rack (Fig. 2b). In Fig. 2a, the piglet is being given a sham injection, while in Fig. 2b, the piglet receives subcutaneous local anesthesia

Handling and restraining devices: We randomized the piglets to be handled with either a commercially available tubular castration bench (TUBE, Fig. 2a) or a custom-made castration rack (HANGING, Fig. 2b). In TUBE, piglets were restrained in a dorsal position in a narrow tubular part, with their hind legs pushed cranially by a metal bar. In HANGING, the piglets were restrained in a vertical position as they were hung from their groins with their heads down while held in place by the handler’s body.

##### Local anesthesia (LA)

**Fig. 3:**
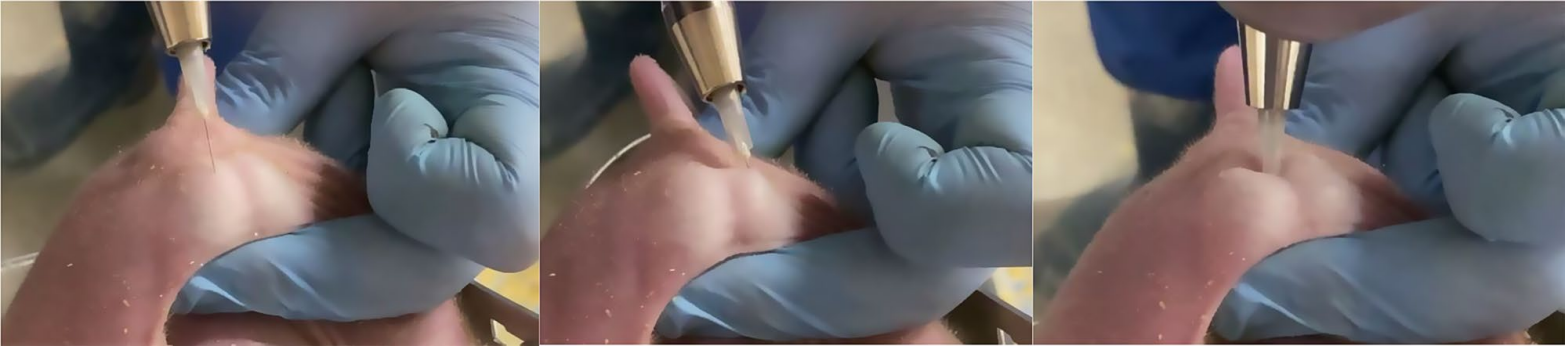
The three-step method of injecting the local anesthetic agent for castration. After securing the testicles with his fingers, the veterinarian inserted the needle subcutaneously on the right testicle, injected the anesthetic agent continuously while withdrawing the needle, released the testicle, and repeated the procedure for the left testis. Lastly, he inserted the needle to its full length (13 mm) between the two testicles, aiming at the area around the spermatic cords, and injected the third dose of the local anesthetic

We randomized half of the TUBE-handled piglets and half of the HANGING-handled piglets to receive either LA or NO LA. We used a solution containing 40 mg/mL procaine hydrochloride and 0.036 mg/mL epinephrine tartrate (Procamidor Comp^®^ Vet., 40 mg/mL, Richter Pharma AG, Wels, Austria), administered the product using an automatic self-filling syringe (HSW ECO-MATIC^®^, Henke-Sass, Wolf GmbH, Tuttlingen, Germany) with a 27 G needle (0.4 × 13 mm) and replaced needles between each litter. For normal-sized piglets, a total of 1.5 mL of the anesthetic solution (equivalent to 60 mg of procaine) was administered across three separate injection sites, with each site receiving 0.5 mL. For small piglets, the dosage was reduced to 0.9 mL in total (equivalent to 36 mg of procaine), divided into three injections of 0.3 mL each. The same veterinarian performed all local anesthetic injections. First, he fixed the testicles caudally between the thumb and middle finger, applying a steady but low pressure during the fixation (see Fig. 3). Then, he inserted the needle just beneath the skin of the scrotum (*Cutis scroti*) of the right testicle and injected the anesthetic agent subcutaneously by continuously dispensing the drug while withdrawing the needle and then releasing the testicle. The veterinarian repeated the same procedure under the scrotal skin of the left testis. He then inserted the needle to its full length (13 mm) between the two testicles, targeting the area around the spermatic cords, and injected the third dose of the local anesthetic agent (Fig. 3). Subsequently, the handler removed the piglet from the restraining device and placed it into the nest.

##### Sham injections (NO LA)

The handler secured the piglets in the restraining devices as described above. The veterinarian then fixed the testicles with his fingers and touched the testicles with an automatic syringe (with a capped needle on) at the same three locations as described in Fig. 3 to simulate LA administration but without any tissue damage. The handler then removed the piglet from the restraining device and placed it into the nest.

##### Castration

After securing the piglet in the restraining device, the handler used a scalpel blade to cut an incision (approximately 1 cm) through the scrotal skin and spermatic fasciae. She then gently pressed the right testicle between her index finger and thumb until it was entirely outside the scrotum. She carefully lifted the testicle vertically and cut the spermatic cord a few millimeters below the testicle using the scalpel. She then made the second incision and repeated the procedure on the left testicle. Between each piglet, she disinfected the blade in a povidone-iodine solution and a new blade was used after each litter.

### Data collection

#### Behavioral reactions

Three observers independently scored piglets’ behavioral reactions (including piglet movements, such as kicking and struggling, and vocalizations) on a scale from 0 (no reaction) to 10 (the worst reaction imaginable) at the following four time points: when the piglet was restrained for the first time (RESTRAIN 1), during local anesthetic injections or sham injections (LOCAL), during the second time the piglet was confined (RESTRAIN 2), and during castration (CASTRATION). Three observers are recommended for such subjective scorings [26]. However, due to space restrictions at the farm, two observers were present during piglet handling and performed direct observations. The third observer assessed the piglets’ reactions from videos recorded with an iPhone placed approximately 20–30 cm from the piglets’ heads. All observers could see with which device the piglet was handled and, for many of the animals, also whether the piglet was receiving LA or NO LA. However, the three observers were blinded regarding whether the piglet was castrated with or without local anesthesia.

#### Vocalizations

We recorded vocalizations from the video recordings described above and analyzed the vocalizations during the administration of LA and NO LA (LOCAL) and during castration (CASTRATION). In the tubular restraining device, both periods started when the handler locked the hind bar that fixed the piglet to the device and ended when she touched the same bar to open it and take the piglet out of the device after injections or castration. In the HANGING restraining device, the periods started when the handler put the piglet’s hind limbs on the device and let go of the legs (i.e., when the piglet was held in place with the handler’s body) and ended when she touched the pig to take it out of the device.

We analyzed the vocalizations with Raven Pro 1.6 bioacoustics analysis software (Cornell Lab of Ornithology, Ithaca, New York, USA). We manually selected each vocalization in the audio files to obtain its duration and categorized it as a grunt, scream, or squeal according to previously reported method [19]. A grunt was characterized as a vocalization with a low tone, a scream as a vocalization with high, long and loud tone (often as long as an expiration of the piglet), and a squeal was a vocalization with high tone (could be with different notes) [19]. We then calculated the mean call durations of all calls and separately for each call type during LOCAL and CASTRATION in seconds. As the durations of different procedures (administering local anesthesia, administering sham injections, and castration) were different, we calculated the duration (seconds) of vocalizations per second of the procedure during LOCAL and CASTRATION by dividing the sum of call durations by the duration of the procedure (obtained from the video recordings).

#### Skin temperature

**Fig. 4:**
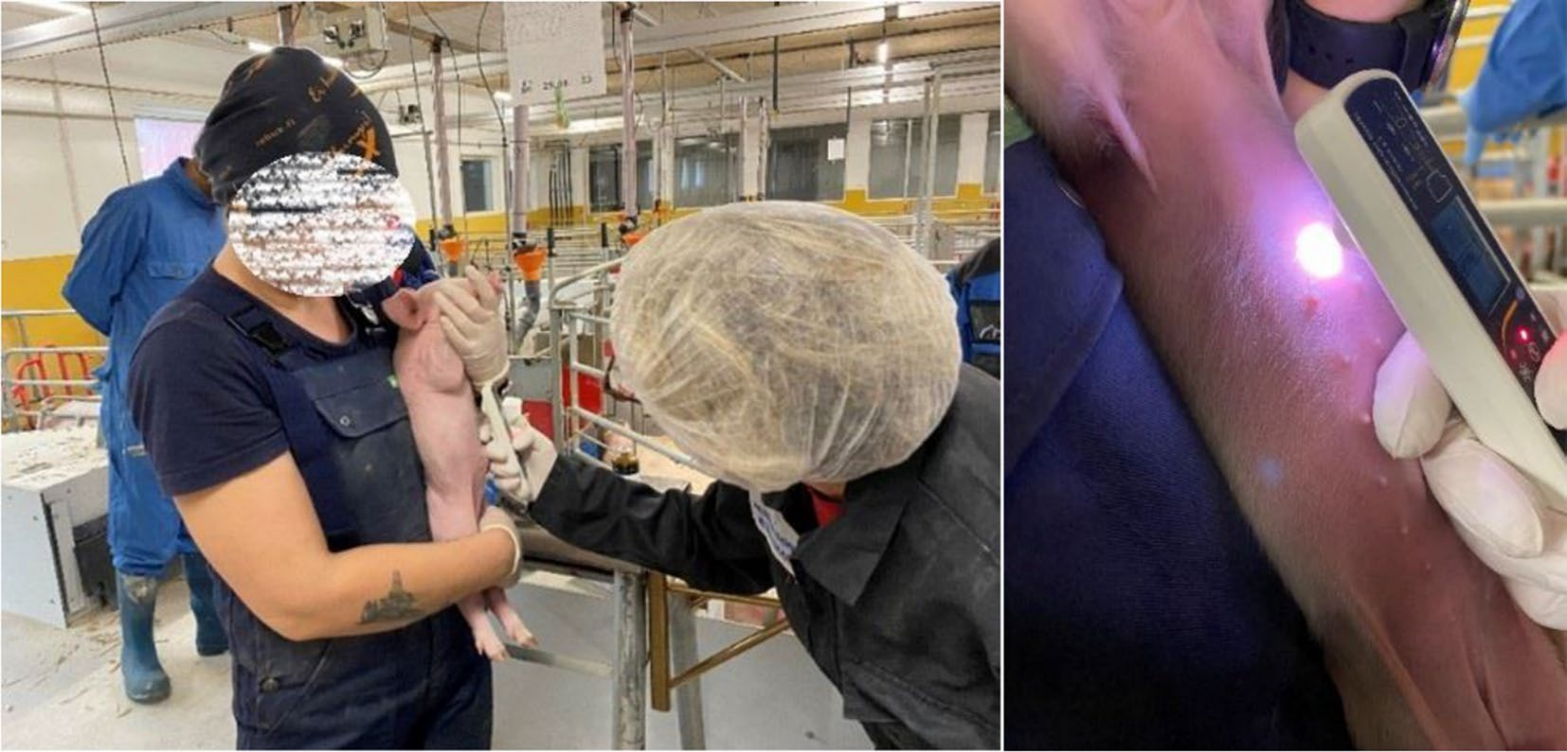
Location of the skin temperature measurement. Skin temperature was measured ventrally at the base of the sternum near the xiphoid process with a hand-held infrared thermometer

We used a hand-held infrared thermometer (model PCE-IR 100; PCE Produktions und Entwicklungsgesellschaft mbH, Meschede, Germany) to measure changes in skin temperature [25]. We measured skin temperature near the internal organs, ventrally at the base of the sternum near the xiphoid process (Fig. 4) after RESTRAIN 1 + LOCAL and RESTRAIN 2 + CASTRATION. Skin temperature was also measured before these procedures to allow us to control individual differences (see also timeline, Fig. 1).

#### Time needed to restrain piglets and duration of procedures

We analyzed video recordings to determine the time required for restraining the piglets (restraining duration) and performing the procedures (administration of local anesthesia, sham injections, and castration) (procedural duration) in the TUBE or HANGING device. For the TUBE, the restraining duration started from the moment the handler turned the piglet onto its back (still in the hands of the handler) and ended when the handler locked the hind bar of the device in the final position. For the HANGING device, the restraining duration started from the moment the handler turned the piglet’s head down in her hands and ended when the piglet’s legs were positioned correctly in the device, and she could let go. The procedural duration (LOCAL or CASTRATION) started when the handler locked the hind bar to its final position (TUBE) or let go of the piglet’s legs (HANGING) and ended when she touched the piglet for the first time to take it out of the device.

### Statistical analysis

We used IBM SPSS Statistics version 29.0 (IBM Inc. Chicago, IL) to analyze the data.

We used an interclass correlation analysis with a two-way mixed effect model assuming consistency to assess the consistency of reaction scoring between the observers. The overall inter-observer reliability was excellent during all four observation periods (Interclass correlation [df = 343–357]: 0.92–0.95, 95% confidence interval: 0.90–94 [lower bound] − 0.93–0.96 [upper bound]). Thus, we used the mean score of the observations in the statistical analysis (mean reaction score). Continuous variables were assessed for normality visually and using the Shapiro-Wilk test.

Mean reaction scores and duration of vocalizations during the four different time points (RESTRAIN 1, LOCAL, RESTRAIN 2, CASTRATION), and skin temperature after the procedures (LOCAL and CASTRATION) were all normally distributed. Accordingly, we studied the effects of the restraining device and local anesthesia on piglets’ mean reaction scores and vocalizations during each of the different time points, and skin temperature after the procedures, using separate mixed models. The models included restraining device (TUBE or HANGING), treatment (local anesthesia [LA] or sham injections [NO LA]), piglet size (small or normal, based on visual estimation), and the interaction between restraining device and treatment as fixed factors, with sow included as a random factor. However, sow as a random factor was redundant in the model for grunts during castration. For temperature after LOCAL and CASTRATION, skin temperature before the procedure was included as a covariate. The results are presented as estimated means (EM) and standard errors (SE). In linear mixed-effects models, deviations from assumption of normality and variance homogeneity were assessed with Kolmogorov-Smirnov test and visually by examining the residual plots.

We studied the time needed to restrain piglets and the duration of different procedures (administration of local anesthesia/sham injections and castration) in the different treatment groups with pairwise comparisons performed with t-tests. The results are presented as mean and standard deviations (SD).

We estimated the sample size based on previous research [3]. Due to technical and human errors, some data were missing (*n* = 172–179 for behavioral reactions and vocalizations, and *n* = 162–176 for skin temperature measurements), resulting in different sample sizes in different comparisons. *P*-values < 0.05 were considered statistically significant and *p*-values < 0.1 as tendencies.

## Results

The 179 piglets were randomized into different treatment groups (LA in TUBE, NO LA in TUBE, LA in HANGING, and NO LA in HANGING), as shown in Table 1. Table 1 also shows the number of piglets per treatment according to their size (normal or small).

**Table 1:** Number of study piglets (*n* = 179) according to their size and treatment group

Treatment group	Piglet size		Total
	normal	small	
Local anesthesia in tubular device	30	19	49
Sham injections in tubular device	24	20	44
Local anesthesia in castration rack	28	15	43
Sham injections in castration rack	31	12	43

### Behavioral reaction scores

**Fig. 5:**
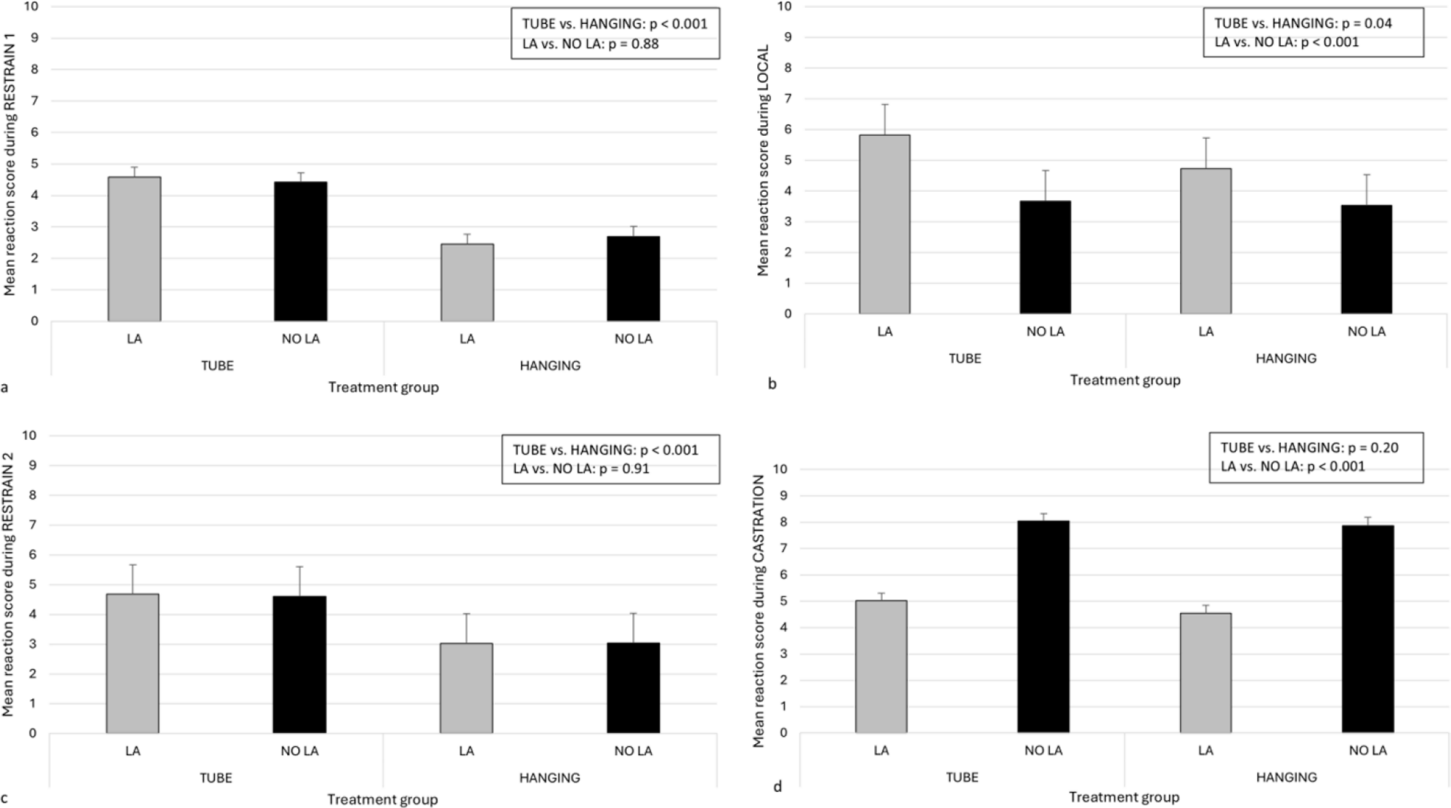
Estimated marginal mean and standard error for mean reaction scores for piglets restrained with different devices and treated with or without local anesthesia. The piglets were restrained either with a tubular device (TUBE) or a castration rack (HANGING). The piglets received either local anesthesia (LA) or sham injections (NO LA) before castration. Three observers scored piglets’ reaction at four time points: (**a**) RESTRAIN 1 (only restraining, *n* = 172); (**b**) LOCAL (restraining and administration of local anesthesia/sham injections, *n* = 177); (**c**) RESTRAIN 2 (only restraining, *n* = 178) and (**d**) CASTRATION (restraining and surgical castration, *n* = 179)

Both the restraining device and the LA vs. NO LA treatment influenced the mean reaction scores of the piglets. As expected, only the device affected the mean reactions during the time points, including handling only (RESTRAIN 1 and RESTRAIN 2, see Fig. 5a and c). The reaction of the piglets was scored to be higher in the TUBE than in the HANGING treatment at both time points. During LOCAL, the reaction of the piglets was scored to be higher both in the TUBE as compared to the HANGING device and in the piglets given LA as compared to NO LA (Fig. 5b). During CASTRATION, the restraining device did not influence the reaction score, but piglets in the NO LA group were scored to react stronger than those in the LA group (Fig. 5d). The interaction between restraining devices and LA vs. NO LA was not significant in any of the models. Small piglets got lower mean reaction scores during all time points compared to normal size piglets (for full model results, see Supplementary Tables 1a-e).

### Vocalizations

**Fig. 6:**
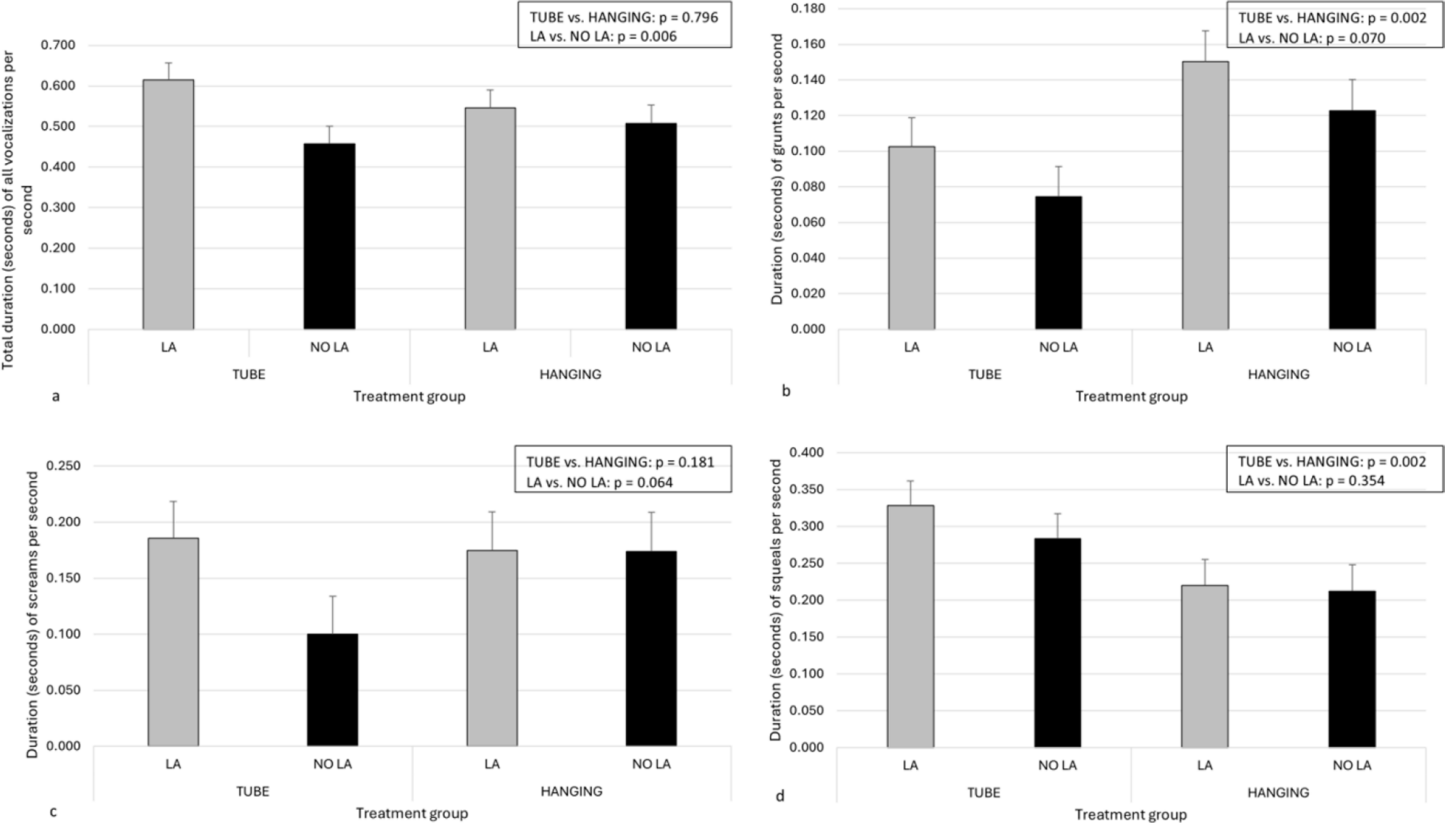
Estimated marginal mean and standard error for the duration (seconds) of piglets’ vocalizations per second during the administration of local anesthesia (LA) or sham injections (NO LA). Different vocalizations of piglets are presented as total vocalizations (**a**, *n* = 178), grunts (**b**, *n* = 178), screams (**c**, *n* = 178), and squeals (**d**, *n* = 178). Piglets had been treated with local anesthesia (LA) or sham injections (NO LA), and they were restrained either with a tubular bench (TUBE) or a castration rack (HANGING)

**Fig. 7:**
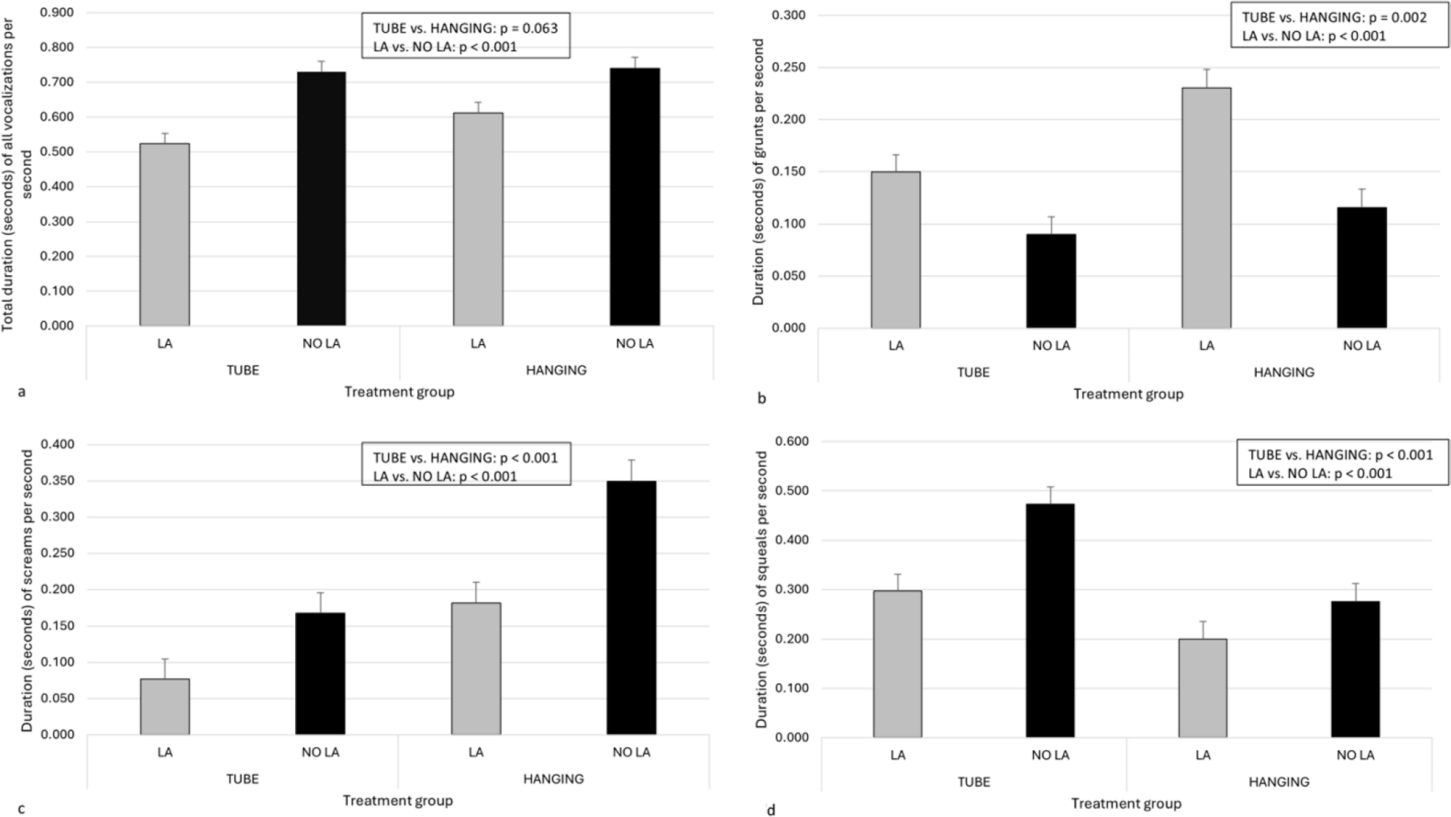
Estimated marginal mean and standard error for duration (seconds) of piglets’ vocalizations per second during castration. Different vocalizations of piglets are presented as total vocalizations (**a**, *n* = 179), grunts (**b**, *n* = 179), screams (**c**, *n* = 179), and squeals (**d**, *n* = 179). Piglets had been treated with local anesthesia (LA) or sham injections (NO LA) and they were restrained either with a tubular bench (TUBE) or a castration rack (HANGING)

Differences in piglets’ vocalizations between the two devices studied and between LA and NO LA are shown in Fig. 6a-d during LOCAL and in Fig. 7a-d during CASTRATION. Overall, piglets that received LA vocalized longer during the administration of local anesthesia (Fig. 6a) and less during castration than piglets in NO LA (Fig. 7a). Moreover, the vocal responses of the piglets during the administration of the local anesthetic (Fig. 6b and d) and castration (Fig. 7b-d) were affected by the restraining device used. The interaction between restraining devices and LA vs. NO LA was not significant in any of the models. There was no difference in the total duration of vocalizations during either LOCAL or CASTRATION between small and normal-sized piglets. However, small piglets grunted longer and screamed less than normal-sized piglets (for full model results, see Supplementary Tables 2a-e and 3a-e).

**Fig. 8:**
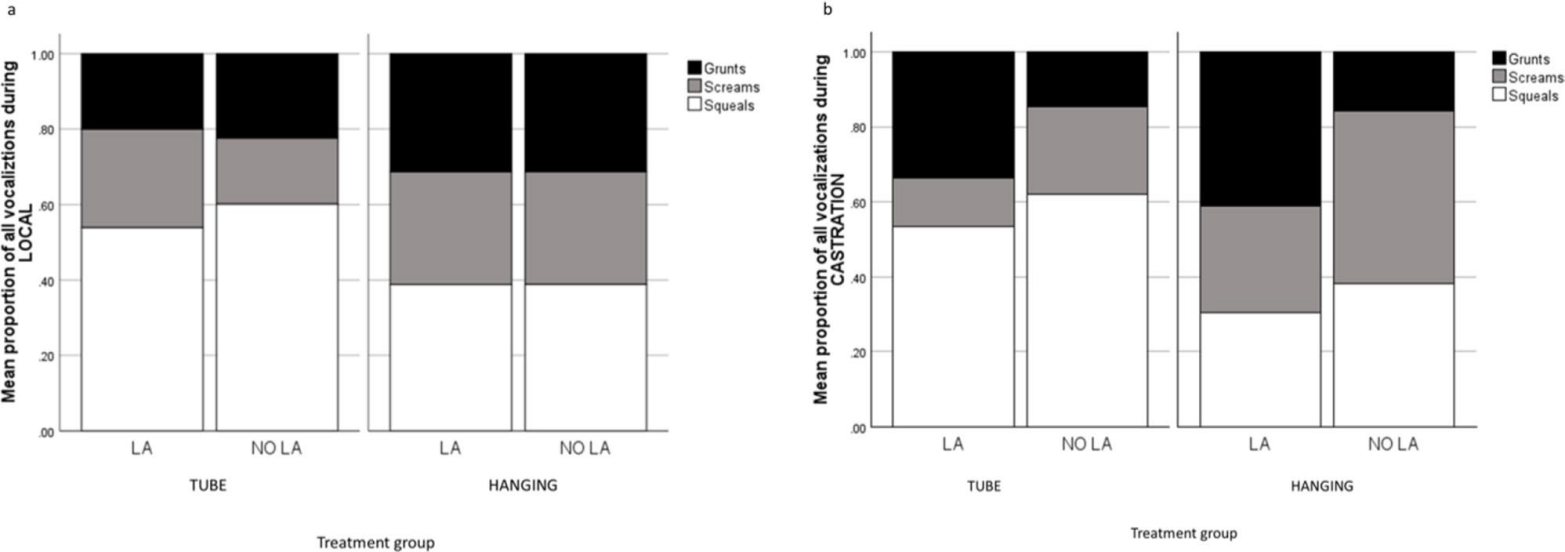
Proportions of different piglet vocalizations of all vocalizations recorded during the procedures. Results are shown for each treatment group separately: the piglets were either restrained in a tubular device (TUBE) or a hanging castration rack (HANGING), and treated with local anesthesia (LA) or with sham injections (NO LA) during administration of local anesthesia or sham injections (LOCAL) (**a**) and during castration (CASTRATION) (**b**)

The proportions of different types of vocalizations (mean durations per second) of piglets in the different treatment groups during LOCAL and CASTRATION are shown in Fig. 8a and b, respectively. A relatively higher proportion of vocalizations were screams during CASTRATION in the piglets that had not received local anesthesia (Fig. 8b). Proportions of grunts and screams were higher in the group restrained in the HANGING device as compared to the TUBE device, while squeals occurred at a higher proportion in the TUBE than in the HANGING handled piglets.

### Skin temperature

**Fig. 9:**
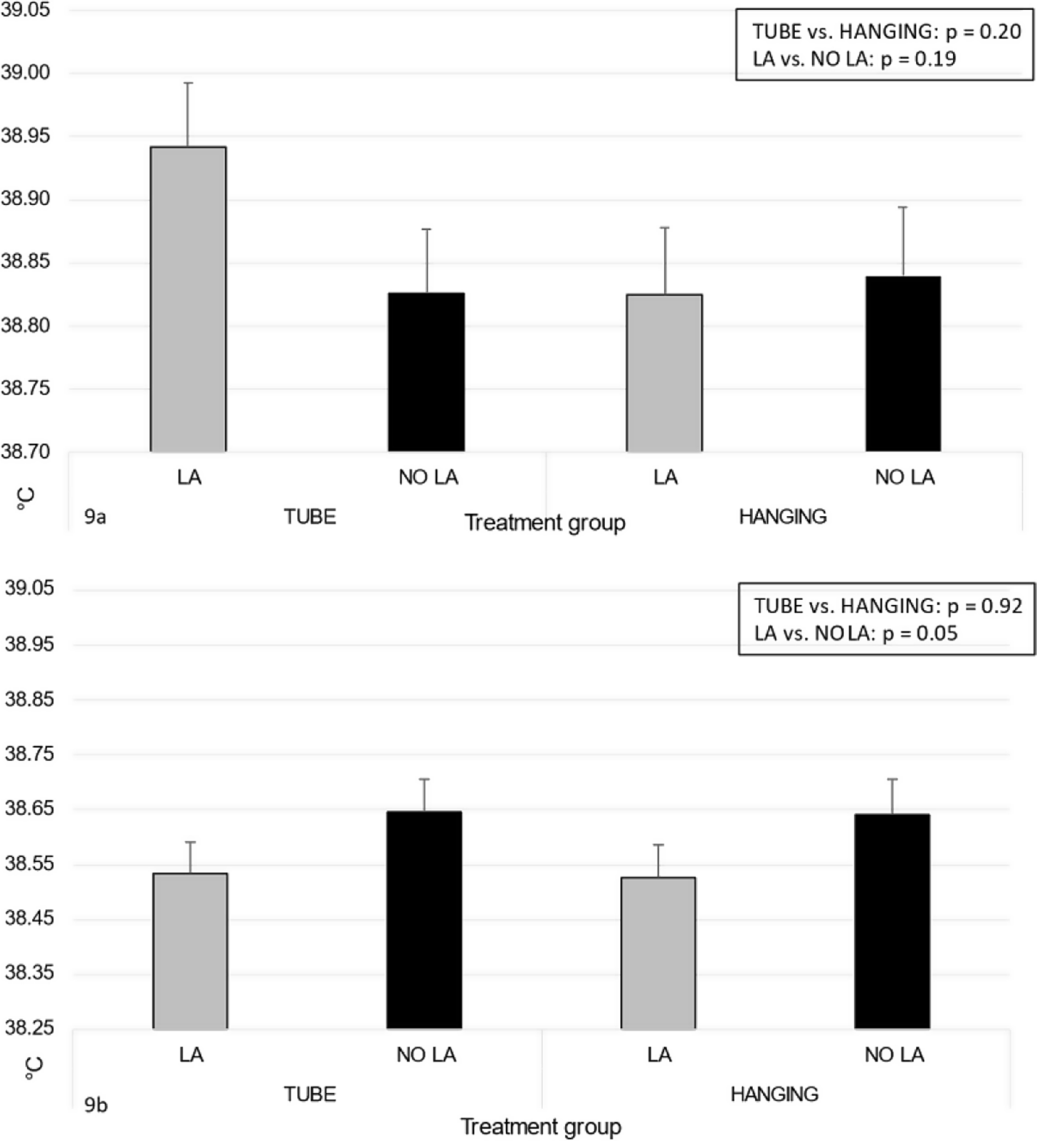
Estimated marginal mean and standard error for skin temperature of piglets measured with an infra-red thermometer near the xiphoid process. Results are shown after the first restraining and administration of local anesthesia (LA) or sham injections (NO LA) (**a**, *n* = 162) and after the second restraining and castration with local anesthesia (LA) or sham injections (NO LA) (**b**, *n* = 176). The piglets were restrained with either a tubular bench (TUBE) or a castration rack (HANGING)

Overall, the skin temperature of the piglets was, on average, 38.9 °C (SD 0.42 °C) before LOCAL and 38.6 °C (0.48 °C) before CASTRATION, and 38.9 °C (0.36 °C) vs. 38.6 °C (0.49 °C) after LOCAL and after CASTRATION respectively. The skin temperature did not differ between the treatment groups after LOCAL, even though it was numerically higher in the TUBE and LA groups than in other treatment groups (Fig. 9a). After CASTRATION, the skin temperature tended to be higher in NO LA piglets than in LA piglets (*p* = 0.05), irrespective of the restraining device used (Fig. 9b). The interaction between the restraining device and LA vs. NO LA was not significant in either of the models. The piglet size category did not influence skin temperature, but temperature before correlated positively with the temperature after both LOCAL and CASTRATION (for full model results, see Supplementary Tables 4a and b).

### Durations required for each procedure

The restraining duration was shorter with HANGING than TUBE (3.5 ± 2.0 versus 6.3 ± 3.2 s, *p* = 0.001). Moreover, administering LA took 14.9 ± 6.1 s, NO LA 9.3 ± 1.6 s, and castration 14.2 ± 4.7 s. Administering LA lasted 11.7 ± 5 s in TUBE and 12.6 ± 5.4 s in HANGING (*p* = 0.928). Castration required 14.3 ± 4.5 s in TUBE and 14.1 ± 4.9 s in HANGING (*p* = 0.843). Castration after LA took less time (13.3 ± 3.1 s) compared to after NO LA (15.2 ± 5.8 s) (*p* < 0.001).

## Discussion

We found that the restraining device affected the piglets’ behavioral reaction scores during handling and piglets’ vocalizations both during local anesthetic administration and castration. Piglets received higher reaction scores when restrained with TUBE than with HANGING. Thus, they possibly perceived TUBE as more aversive than HANGING. The validity of our reaction scoring method is supported by the piglets’ stronger behavioral reaction scores during local anesthetic administration (LA) than the sham injections (NO LA). Moreover, NO LA piglets had higher reaction scores during castration than LA piglets. Our findings are consistent with the literature, as several authors concluded that local anesthesia induces stress, discomfort, and pain [3, 4, 27], and piglets treated with local anesthesia prior to castration resisted less during the procedure than their counterparts castrated without local anesthesia [2, 11, 28].

Interpreting the results becomes more complex when vocalizations are considered; piglets tended to vocalize longer when castrated in HANGING than in TUBE. This contradicts our finding that piglets had higher behavioral scores when restrained in TUBE. Indeed, piglets also vocalized longer when receiving LA than NO LA and when castrated after NO LA than LA. These findings are consistent with other studies [3, 4, 29, 30]. For example, Coutant et al. [3] found that the local anesthetic injections resulted in higher values of vocal parameters than those of sham-handled piglets, indicating a significant stress response [31]. Moreover, Lou et al. [30] observed that piglets castrated under 24 h of age produced more vocalizations of a higher frequency than sham-castrated piglets, and these high-frequency vocalizations tended to be longer in duration during castration than during sham castration. In contrast to our findings, Weary and colleagues [21] found no effect of the handling method on the rate of high calls produced by the piglets in connection to castration. Piglets suspended by their rear legs in the handler’s hands had low calls at a slower rate than piglets restrained in a bench in a dorsal position. Moreover, piglets restrained dorsally on the bench produced low calls at a lower rate than those restrained in a V-trough [21]. Thus, evaluating different restraining methods by their aversiveness needs further research. It is possible that longer vocalizations produced while castrated with HANGING when compared with TUBE may result from different body postures during restraining; piglets could probably vocalize longer while hanging upside down than when restrained in the dorsal position with the hind legs pushed cranially. Additionally, it is also possible that the two different restraining postures were perceived as differentially aversive. Being restrained dorsally in a narrow TUBE and having legs fixated on the bench may provoke more resistance behavior as an attempt to escape and adjust body postures. However, being hung upside down could induce more fear of falling, thus resulting in fewer resistance movements but more and longer high-tone vocalizations, such as screams, which are often associated with fear, panic, and pain [22].

The restraining device, administration of local anesthetic, and castration all affected the durations and proportions of different vocalizations in piglets. During LOCAL, the total duration of all vocalizations was significantly longer in LA than in NO LA. Furthermore, although the restraining device did not influence the duration of all vocalizations during LOCAL, the piglets grunted longer when restrained with HANGING and squealed longer in TUBE. During castration, the total duration of all vocalizations was longer among NO LA piglets than those castrated with LA. Moreover, the restraining device influenced the duration of all vocalizations during castration, as piglets tended to vocalize for a longer time in HANGING than in TUBE. Specifically, the piglets’ grunts and screams were longer in HANGING than in TUBE, and piglets squealed longer in TUBE. Interestingly, the restraining device and the severity or type of pain experienced by piglets appear to affect the different proportions of vocalizations during painful procedures. During LOCAL, the proportion of screams was greater among LA piglets than among NO LA piglets in TUBE but not in HANGING. In contrast, the proportion of grunts remained similar in both devices, while the proportion of squeals was slightly greater among NO LA than among LA piglets in TUBE but again not in HANGING. In contrast, the vocalization pattern shifted noticeably during castration; the proportion of grunts was much lower, and the proportions of screams and squeals were much greater among NO LA than LA. The proportion of screams was especially high when piglets were restrained in the vertical position during castration. Thus, grunting could be more a sign of overall discomfort (and maybe also a piglet’s effort to contact the mother sow) and is replaced by squealing and screaming, calls previously found to be used by piglets experiencing severe pain and distress [11, 21, 22, 32], during castration.

We wanted to study piglets’ reactions and vocalizations in a real-life setting in a commercial piggery with as few deviations from normal procedures as possible. Naturally, this approach limited our ability to record more advanced vocal parameters. There are limitations in using durations and categories of vocalizations to measure pain and stress in young piglets. Indeed, previous studies have shown that more advanced vocal parameters, such as frequency, energy, or amplitude, seem to be more sensitive and accurate in reporting pain and stress than other general measures, such as average call duration or proportion of call time used in the current study [19]. Thus, future work could focus on these more complex indicators of pain and distress. Moreover, future studies are needed to understand how the physical situation restricts piglets’ ability to express their emotions and experiences with different types of vocalizations. Finally, our results indicate that the size of the piglet affects their vocalizations and should be considered with more care in future studies.

We chose to use vocalization variables standardized for time (i.e., duration per second), as the different time points during the experiment differed in duration within and between treatments. Thus, the outcome reflects the *intensity* of the negative effect of the procedures on the piglets. However, from a welfare perspective, it may be even more relevant to assess the *magnitude* of the impact, i.e., the combination of intensity and duration [33]. For this, we could have used the total duration of vocalizations (without standardizing for timepoint duration) as the outcome variables. To test for this, we analyzed the results with both types of variables, while we decided only to report the intensity results here for simplicity. The results were very similar when using intensity and when using magnitude variables. As we believe the intensity results are easier to compare with other studies using the same approach [e.g., 19], we opted only to report these.

We expected the skin temperature would increase while resisting procedures by trying to escape, kicking, and vocalizing. Moreover, as our measuring point is near the internal organs and the activation of the sympathetic nervous system causes vasoconstriction and redirection of blood flow to the internal organs [23], an increase in temperature is expected. Indeed, the mean skin temperatures were higher after castration with NO LA than with LA. This aligns with our findings that the mean behavioral reaction scores were greater among piglets in the NO LA compared with the LA treatment group during CASTRATION. We chose this method for measuring skin temperature to add a non-invasive, rapid, and objective measurement indicative of piglets’ stress and resistance movements to our study design. We acknowledge that our approach of using changes in skin temperature as an indicator of stress and pain differs from the approach in previous research. Specifically, prior research considered a decrease in skin surface temperature, measured in the groin area following castration, as an indicator of sympathetic nervous system activation and, thus, distress and pain [25]. Future research should be conducted to explore how the skin temperature measured near the xiphoid process and other sites of the body correlates with the actual body temperature among young piglets experiencing stress and pain while restrained in different postures. This would build on suggestions of previous studies examining castration-related pain in piglets [11] and reviews on the use of infra-red skin temperature measurement for monitoring porcine health and assessing pain [1, 24].

In addition to evaluating piglets’ behavioral reactions, vocalizations, and skin temperature, our results revealed that these restraining devices also differed in handling duration. More time was needed to restrain the piglets in TUBE than HANGING. However, the time needed to administer LA was similar between the devices. There were also no differences between the two handling methods in the duration of the castration procedure. It is worth noting that our operator has long experience in castrating, but both devices were new to her as she usually performs the procedure by holding piglets between her thighs. However, our results show that castrating piglets with LA took less time than performing the procedure after NO LA, consistent with findings from previous research [2]. Considering the time needed to handle and restrain piglets is essential, as shorter handling times are preferred for reasons of animal welfare and time management. Moreover, as using local anesthesia approximately doubles the overall time needed to castrate individual piglets, the handling method should allow quick injections. Therefore, our results emphasize the need to develop easy-to-use methods that minimize stress for the piglets while also facilitating procedures and ensuring safety for the operator. Moreover, as strong reactions to handling could potentially hinder caregivers’ ability to accurately assess the methods used to provide local anesthesia before castration and the efficacy of local anesthesia during castration, less stressful handling methods are needed both in scientific research and in practice. Thus, future studies are warranted on how piglets react to handling, restraining, administration of local anesthesia, and castration while being held in the assistant’s lap.

The optimal technique to administer local anesthesia in young piglets for castration remains unknown [1, 3, 34]. In particular, intratesticular injections, while common, cause pain and possible tissue damage [3, 4]. To address this, we employed a modified technique designed to deliver the local anesthetic as close as possible to the incision sites but under the skin and deeper to the area where the spermatic cords will be cut. However, consistent with previous findings, LA still resulted in significant behavioral reactions compared with NO LA [3, 4]. Thus, the efficacy of local anesthesia before piglet castration in improving welfare remains a subject of ongoing debate. The benefits of pain reduction during castration must be weighed against the stress and pain associated with its administration. Further research is needed to optimize anesthetic protocols, refine handling techniques, and develop alternative methods that minimize both the procedural and post-procedural pain experienced by piglets.

One of our aims was to assess whether the restraining device hinders the effective evaluation of the local anesthesia protocol during castration, as it is essential that the operator can make a reliable assessment of the efficacy of the local anesthesia used. Our results suggest that the type of restraining device used may indeed hinder this assessment. For instance, piglets restrained in the tubular device received almost similar mean behavioral scores both when first restrained and during castration under local anesthesia. Notably, the mean difference in behavioral scores between piglets castrated with or without local anesthesia was 3 points on a 0 to 10 scale, which is just one point more than the difference in scores observed between the two restraining devices during the initial restraint. This indicates that strong reaction to handling can make it challenging, if not impossible, to reliably assess the efficacy of local anesthesia.

## Conclusion

The choice of restraining method significantly affects piglets’ reactions and vocalizations, potentially preventing an accurate assessment of local anesthesia efficacy. However, neither device was optimal as both induced strong reactions among the piglets. It is difficult to compare the devices based on our results, especially as they seem to affect the types of vocalizations in a manner we cannot fully explain. Moreover, while the administration of procaine with epinephrine effectively alleviates pain in piglets aged 3 to 4 days, it also requires additional handling and needle injections, inducing stress and pain, which is evident through stronger reactions and longer and more intense vocalizations. Besides increasing the welfare burden of the procedure for the piglets, the strong reactions to handling can complicate or even prevent the assessment of pain and distress caused by the local anesthetic injections and the effect of local anesthesia during castration. Therefore, our research underscores the importance of identifying the best methods for restraining young piglets for local anesthesia administration and castration.

## Electronic supplementary material

Below is the link to the electronic supplementary material.

Supplementary Material 1

## Data Availability

The datasets generated and/or analyzed during the current study are not publicly available due to being under further analysis but are available from the corresponding author upon reasonable request.
