# Supplementary material for: Two restraining devices in connection to surgical castration with or without local anesthesia: effects on piglet stress
Source: Porcine Health Manag. 2025 Apr 15;11:21. doi: 10.1186/s40813-025-00428-7 (PMC12001485; doi:10.1186/s40813-025-00428-7)
Supplement: Supplementary file 1 — Supplementary Material 1 [file 40813_2025_428_MOESM1_ESM.docx]

Supplementary Tables

Table 1a Linear mixed model output for mean behavioral reactions for piglets during the first restraint.

|  | df | F | p-value |
| --- | --- | --- | --- |
| Intercept | 23.6 | 225.7 | < 0.001 |
| Restraining device^1)^ | 149.1 | 65.9 | < 0.001 |
| Local anesthesia^2)^ | 147.5 | 0.02 | 0.9 |
| Restraining device*Local anesthesia^3)^ | 148.9 | 0.8 | 0.4 |
| Size^4)^ | 116.0 | 17.9 | < 0.001 |

Number of subjects (piglets) in the model was 172. ^1)^ Restraining device was either tubular device (TUBE) or a castration rack (HANGING). ^2)^ Piglets received either local anesthesia (LA) or sham injections (NO LA). ^3)^ The interaction of restraining device and local anesthesia was included in the model. ^4)^ The size of the piglets was visually estimated to be either normal or small.

**Table 1b** Linear mixed model output for mean behavioral reactions for piglets during administration of local anesthesia.

|  | df | F | p-value |
| --- | --- | --- | --- |
| Intercept | 22.7 | 238.5 | < 0.001 |
| Restraining device^1)^ | 153.6 | 4.5 | 0.04 |
| Local anesthesia^2)^ | 151.7 | 34.5 | < 0.001 |
| Restraining device*Local anesthesia^3)^ | 153.2 | 2.7 | 0.1 |
| Size^4)^ | 112.2 | 6.4 | 0.01 |

Number of subjects (piglets) in the model was 177. ^1)^ Restraining device was either tubular device (TUBE) or a castration rack (HANGING). ^2)^ Piglets received either local anesthesia (LA) or sham injections (NO LA). ^3)^ The interaction of restraining device and local anesthesia was included in the model. ^4)^ The size of the piglets was visually estimated to be either normal or small.

Table 1c Linear mixed model output for mean behavioral reactions for piglets during the second restraint.

|  | df | F | p-value |
| --- | --- | --- | --- |
| Intercept | 22.4 | 362.2 | < 0.001 |
| Restraining device^1)^ | 155.0 | 50.2 | < 0.001 |
| Local anesthesia^2)^ | 152.7 | 0.01 | 0.9 |
| Restraining device*Local anesthesia^3)^ | 154.4 | 0.03 | 0.9 |
| Size^4)^ | 92.9 | 19.7 | < 0.001 |

Number of subjects (piglets) in the model was 178. ^1)^ Restraining device was either tubular device (TUBE) or a castration rack (HANGING). ^2)^ Piglets received either local anesthesia (LA) or sham injections (NO LA). ^3)^ The interaction of restraining device and local anesthesia was included in the model. ^4)^ The size of the piglets was visually estimated to be either normal or small.

Table 1d Linear mixed model output for mean behavioral reactions for piglets during castration.

|  | df | F | p-value |
| --- | --- | --- | --- |
| Intercept | 20.6 | 1066.0 | < 0.001 |
| Restraining device^1)^ | 155.7 | 1.6 | 0.2 |
| Local anesthesia^2)^ | 152.7 | 158.6 | < 0.001 |
| Restraining device*Local anesthesia^3)^ | 154.6 | 0.4 | 0.5 |
| Size^4)^ | 68.4 | 9.5 | 0.003 |

Number of subjects (piglets) in the model was 179. ^1)^ Restraining device was either tubular device (TUBE) or a castration rack (HANGING). ^2)^ Piglets received either local anesthesia (LA) or sham injections (NO LA). ^3)^ The interaction of restraining device and local anesthesia was included in the model. ^4)^ The size of the piglets was visually estimated to be either normal or small.

Table 1e Estimated marginal means and standards errors for mean reaction scores for piglets divided into two size groups (normal and small).

| Observation timepoint | Number of the piglets in the model | Size of the piglets | EM | SE | p-value |
| --- | --- | --- | --- | --- | --- |
| Mean behavioral reactions for piglets during the first restraint | 172 | Normal  Small | 4.3  2.8 | 0.3  0.3 | < 0.001 |
| Mean behavioral reactions for piglets during administration of local anesthesia | 177 | Normal  Small | 5.0  3.9 | 0.3  0.4 | 0.01 |
| Mean behavioral reactions for piglets during the second restraint | 178 | Normal  Small | 4.6  3.1 | 0.2  0.3 | < 0.001 |
| Mean behavioral reactions for piglets during castration | 179 | Normal  Small | 6.9  5.9 | 0.2  0.3 | 0.003 |

The size of the piglets was visually estimated to be either normal or small. The piglets were restrained either with a tubular device (TUBE) or a castration rack (HANGING). The piglets received either local anesthesia (LA) or sham injections (NO LA) before castration. Their reaction was scored by three observers at four timepoints: the first restraint (RESTRAIN 1, only restraining), during administration of local anesthesia (LOCAL, restraining and administration of local anesthesia/sham injections), during the second restraint (RESTRAIN 2, only restraining), and during castration (CASTRATION, restraining and surgical castration).

**Table 2a** Linear mixed model output for total duration of all vocalizations per second during LOCAL.

|  | df | F | p-value |
| --- | --- | --- | --- |
| Intercept | 23.6 | 290.2 | < 0.001 |
| Restraining device^1)^ | 156.1 | 0.07 | 0.8 |
| Local anesthesia^2)^ | 153.8 | 7.8 | 0.006 |
| Restraining device*Local anesthesia^3)^ | 155.3 | 2.9 | 0.09 |
| Size^4)^ | 95.1 | 1.2 | 0.3 |

Number of subjects (piglets) in the model was 178. ^1)^ Restraining device was either tubular device (TUBE) or a castration rack (HANGING). ^2)^ Piglets received either local anesthesia (LA) or sham injections (NO LA). ^3)^ The interaction of restraining device and local anesthesia was included in the model. ^4)^ The size of the piglets was visually estimated to be either normal or small.

**Table 2b** Linear mixed model output for duration of grunts per second during LOCAL.

|  | df | F | p-value |
| --- | --- | --- | --- |
| Intercept | 23.5 | 114.7 | < 0.001 |
| Restraining device^1)^ | 158.1 | 9.7 | 0.002 |
| Local anesthesia^2)^ | 155.3 | 3.3 | 0.07 |
| Restraining device*Local anesthesia^3)^ | 156.8 | 0.001 | 1.0 |
| Size^4)^ | 61.9 | 5.1 | 0.03 |

Number of subjects (piglets) in the model was 178. ^1)^ Restraining device was either tubular device (TUBE) or a castration rack (HANGING). ^2)^ Piglets received either local anesthesia (LA) or sham injections (NO LA). ^3)^ The interaction of restraining device and local anesthesia was included in the model. ^4)^ The size of the piglets was visually estimated to be either normal or small.

**Table 2c** Linear mixed model output for duration of screams per second during LOCAL.

|  | df | F | p-value |
| --- | --- | --- | --- |
| Intercept | 22.2 | 33.2 | < 0.001 |
| Restraining device^1)^ | 153.3 | 1.8 | 0.2 |
| Local anesthesia^2)^ | 151.6 | 3.5 | 0.06 |
| Restraining device*Local anesthesia^3)^ | 152.9 | 3.3 | 0.07 |
| Size^4)^ | 138.1 | 4.6 | 0.03 |

Number of subjects (piglets) in the model was 178. ^1)^ Restraining device was either tubular device (TUBE) or a castration rack (HANGING). ^2)^ Piglets received either local anesthesia (LA) or sham injections (NO LA). ^3)^ The interaction of restraining device and local anesthesia was included in the model. ^4)^ The size of the piglets was visually estimated to be either normal or small.

**Table 2d** Linear mixed model output for duration of squeals per second during LOCAL.

|  | df | F | p-value |
| --- | --- | --- | --- |
| Intercept | 22.0 | 118.8 | < 0.001 |
| Restraining device^1)^ | 155.3 | 9.6 | 0.002 |
| Local anesthesia^2)^ | 152.7 | 0.9 | 0.4 |
| Restraining device*Local anesthesia^3)^ | 154.3 | 0.4 | 0.5 |
| Size^4)^ | 82.7 | 0.2 | 0.6 |

Number of subjects (piglets) in the model was 178. ^1)^ Restraining device was either tubular device (TUBE) or a castration rack (HANGING). ^2)^ Piglets received either local anesthesia (LA) or sham injections (NO LA). ^3)^ The interaction of restraining device and local anesthesia was included in the model. ^4)^ The size of the piglets was visually estimated to be either normal or small.

Table 2e Estimated marginal means and standards errors for durations (seconds) of piglets’ vocalizations per second during (LOCAL).

| Vocalizations | Number of the piglets in the model | Size of the piglets | EM | SE | p-value |
| --- | --- | --- | --- | --- | --- |
| Total vocalizations | 178 | Normal  Small | 0.6  0.5 | 0.04  0.04 | 0.3 |
| Grunts | 178 | Normal  Small | 0.09  0.1 | 0.01  0.02 | 0.03 |
| Screams | 178 | Normal  Small | 0.2  0.1 | 0.03  0.04 | 0.03 |
| Squeals | 178 | Normal  Small | 0.3  0.3 | 0.03  0.03 | 0.6 |

The size of the piglets was visually estimated to be either normal or small. The piglets were restrained either with a tubular device (TUBE) or a castration rack (HANGING). The piglets received either local anesthesia (LA) or sham injections (NO LA) before castration. Different vocalizations of piglets (total vocalizations, grunts, screams, and squeals) were analyzed during local anesthetic injections or sham injections (LOCAL).

**Table 3a** Linear mixed model output for total duration of all vocalizations per second during CASTRATION.

|  | df | F | p-value |
| --- | --- | --- | --- |
| Intercept | 21.5 | 1111.5 | < 0.001 |
| Restraining device^1)^ | 156.8 | 3.5 | 0.06 |
| Local anesthesia^2)^ | 153.8 | 41.2 | < 0.001 |
| Restraining device*Local anesthesia^3)^ | 155.6 | 2.1 | 0.1 |
| Size^4)^ | 66.5 | 2.0 | 0.2 |

Number of subjects (piglets) in the model was 179. ^1)^ Restraining device was either tubular device (TUBE) or a castration rack (HANGING). ^2)^ Piglets received either local anesthesia (LA) or sham injections (NO LA). ^3)^ The interaction of restraining device and local anesthesia was included in the model. ^4)^ The size of the piglets was visually estimated to be either normal or small.

**Table 3b** Linear mixed model output for duration of grunts per second during CASTRATION.

|  | df | F | p-value |
| --- | --- | --- | --- |
| Intercept | 174 | 275.5 | < 0.001 |
| Restraining device^1)^ | 174 | 9.7 | 0.002 |
| Local anesthesia^2)^ | 174 | 26.5 | < 0.001 |
| Restraining device*Local anesthesia^3)^ | 174 | 2.6 | 0.1 |
| Size^4)^ | 174 | 5.0 | 0.03 |

Number of subjects (piglets) in the model was 179. ^1)^ Restraining device was either tubular device (TUBE) or a castration rack (HANGING). ^2)^ Piglets received either local anesthesia (LA) or sham injections (NO LA). ^3)^ The interaction of restraining device and local anesthesia was included in the model. ^4)^ The size of the piglets was visually estimated to be either normal or small.

**Table 3c** Linear mixed model output for duration of screams per second during CASTRATION.

|  | df | F | p-value |
| --- | --- | --- | --- |
| Intercept | 20.2 | 97.5 | < 0.001 |
| Restraining device^1)^ | 154.8 | 35.3 | < 0.001 |
| Local anesthesia^2)^ | 151.9 | 29.2 | < 0.001 |
| Restraining device*Local anesthesia^3)^ | 153.8 | 2.6 | 0.1 |
| Size^4)^ | 74.9 | 3.0 | 0.09 |

Number of subjects (piglets) in the model was 179. ^1)^ Restraining device was either tubular device (TUBE) or a castration rack (HANGING). ^2)^ Piglets received either local anesthesia (LA) or sham injections (NO LA). ^3)^ The interaction of restraining device and local anesthesia was included in the model. ^4)^ The size of the piglets was visually estimated to be either normal or small.

**Table 3d** Linear mixed model output for duration of squeals per second during CASTRATION.

|  | df | F | p-value |
| --- | --- | --- | --- |
| Intercept | 21.6 | 153.4 | < 0.001 |
| Restraining device^1)^ | 155.6 | 25.4 | < 0.001 |
| Local anesthesia^2)^ | 153.0 | 18.9 | < 0.001 |
| Restraining device*Local anesthesia^3)^ | 154.8 | 3.0 | 0.08 |
| Size^4)^ | 86.4 | 0.2 | 0.6 |

Number of subjects (piglets) in the model was 179. ^1)^ Restraining device was either tubular device (TUBE) or a castration rack (HANGING). ^2)^ Piglets received either local anesthesia (LA) or sham injections (NO LA). ^3)^ The interaction of restraining device and local anesthesia was included in the model. ^4)^ The size of the piglets was visually estimated to be either normal or small.

Table 3e Estimated marginal means and standards errors for durations (seconds) of piglets’ vocalizations per second during CASTRATION.

| Vocalizations | Number of the piglets in the model | Size of the piglets | EM | SE | p-value |
| --- | --- | --- | --- | --- | --- |
| Total vocalizations | 179 | Normal  Small | 0.7  0.6 | 0.02  0.03 | 0.2 |
| Grunts | 179 | Normal  Small | 0.1  0.2 | 0.01  0.01 | 0.03 |
| Screams | 179 | Normal  Small | 0.2  0.2 | 0.02  0.03 | 0.09 |
| Squeals | 179 | Normal  Small | 0.3  0.3 | 0.03  0.04 | 0.6 |

The size of the piglets was visually estimated to be either normal or small. The piglets were restrained either with a tubular device (TUBE) or a castration rack (HANGING). The piglets received either local anesthesia (LA) or sham injections (NO LA) before castration. Different vocalizations of piglets (total vocalizations, grunts, screams, and squeals) were analyzed during castration (CASTRATION).

**Table 4a** Linear mixed model output for skin temperature after LOCAL.

|  | df | F | p-value |
| --- | --- | --- | --- |
| Intercept | 158.7 | 88.6 | < 0.001 |
| Restraining device^1)^ | 141.9 | 1.7 | 0.2 |
| Local anesthesia^2)^ | 137.6 | 1.7 | 0.2 |
| Restraining device*Local anesthesia^3)^ | 138.5 | 2.9 | 0.09 |
| Skin temperature before^4)^ | 158.8 | 73.5 | < 0.001 |
| Size^5)^ | 111.2 | 0.4 | 0.5 |

Number of subjects (piglets) in the model was 162. ^1)^ Restraining device was either tubular device (TUBE) or a castration rack (HANGING). ^2)^ Piglets received either local anesthesia (LA) or sham injections (NO LA). ^3)^ The interaction of restraining device and local anesthesia was included in the model. ^4)^ The skin temperature before local anesthetic or sham injections was measured with infra-red thermometer near the xiphoid process. ^5)^ The size of the piglets was visually estimated to be either normal or small.

Table 4b Linear mixed model output for skin temperature after CASTRATION.

|  | df | F | p-value |
| --- | --- | --- | --- |
| Intercept | 161.9 | 36.3 | < 0.001 |
| Restraining device^1)^ | 151.0 | 0.01 | 0.9 |
| Local anesthesia^2)^ | 146.8 | 3.8 | 0.05 |
| Restraining device*Local anesthesia^3)^ | 147.3 | 0.002 | 1.0 |
| Skin temperature before^4)^ | 162.1 | 93.5 | < 0.001 |
| Size^5)^ | 42.0 | 0.01 | 0.9 |

Number of subjects (piglets) in the model was 176. ^1)^ Restraining device was either tubular device (TUBE) or a castration rack (HANGING). ^2)^ Piglets received either local anesthesia (LA) or sham injections (NO LA). ^3)^ The interaction of restraining device and local anesthesia was included in the model. ^4)^ The skin temperature before castration was measured with infra-red thermometer near the xiphoid process. ^5)^ The size of the piglets was visually estimated to be either normal or small.

Table 4c Estimated marginal means and standards errors for skin temperature after LOCAL and CASTRATION.

|  | Number of the piglets in the model | Size of the piglets | EM | SE | p-value |
| --- | --- | --- | --- | --- | --- |
| After LOCAL | 162 | Normal  Small | 38.9  38.8 | 0.04  0.06 | 0.5 |
| After CASTRATION | 176 | Normal  Small | 38.6  38.6 | 0.04  0.06 | 0.9 |

The size of the piglets was visually estimated to be either normal or small. The piglets were restrained either with a tubular device (TUBE) or a castration rack (HANGING). The piglets received either local anesthesia (LA) or sham injections (NO LA) before castration. The skin temperature before and after local anesthesia administration or sham injections and before and after castration was measured with infra-red thermometer near the xiphoid process.
